# Characterizing Media Content and Effects of Organ Donation on a Social Media Platform: Content Analysis

**DOI:** 10.2196/13058

**Published:** 2019-03-12

**Authors:** Xiaoya Jiang, Wenshi Jiang, Jiawei Cai, Qingdong Su, Zhigang Zhou, Lingnan He, Kaisheng Lai

**Affiliations:** 1 School of Communication and Design Sun Yat-Sen University Guangzhou China; 2 Intelligence Sharing for Life Science Research Institute Shenzhen China; 3 923 Hospital of Chinese People's Liberation Army Nanning China; 4 The First People's Hospital of Kunming Kunming China; 5 Guangdong Key Laboratory for Big Data Analysis and Simulation of Public Opinion Guangzhou China; 6 School of Journalism and Communication Jinan University Guangzhou China

**Keywords:** organ donation, media, media effects, social media

## Abstract

**Background:**

The lack of organ donors has become a barrier for the development of organ transplantation programs, and many countries are currently facing a severe shortage of deceased organs. Media campaigns on social media have the potential to promote organ donation. However, little is known about what kind of media content is the most appropriate for this purpose.

**Objective:**

This study aimed to analyze media posts regarding organ donation on Weibo, a social media platform, and to identify the media themes that are most advantageous in promoting public awareness and attitudes concerning organ donation.

**Methods:**

Based on 16 million social media users’ posts randomly extracted from January 1 to December 31, 2017, 1507 reposts of 141 distinct media posts relevant to organ donation were found. We analyzed the media posts’ themes and examined their effects in promoting public awareness about organ donation by comparing the number of reposts and comments they prompted. The themes’ impact on attitude toward organ donation was gauged using the comments indicating support and intentions for organ donation.

**Results:**

Overall, 5 major themes were identified from the media posts, among which “organ donation behaviors” constituted the highest proportion (58/141, 41.13%). However, themes of “statistical descriptions of organ donation” and “meaningfulness of donation” were the most influential in promoting awareness on organ donation: approximately 3 of 10 commenters for the former theme and 2 of 10 commenters for the latter expressed intentions to become organ donors. These two themes, along with “meaningfulness of organ donation for society,” a subtheme of “meaningfulness of donation,” were the most effective for evoking support and intentions for donation.

**Conclusions:**

A discrepancy was revealed between the media themes that were the most salient on the media agenda and those that were the most effective in increasing organ donation awareness and intentions on social media. These findings provide guidance for campaigns on organ donation. The results also suggest the potential of campaigns on social media for promoting prosocial health behaviors and highlight the importance of strategic message design for serving this goal.

## Introduction

### Background

Organ transplantation has brought hope to people with illness previously considered incurable [1]. However, the donated organ is a prerequisite of organ transplantation, and the need for organ donation has increased globally in the past years [2]. In China, for every 30 patients requiring organ transplantation, only one receives the organ [3]. Given the alarming extent of shortage of organ donation, measures have been actively taken to promote donation among citizens through policy making and simplification of procedure. In 2013, the National Health Commission of China announced the Provisions on the Human Organ Procurement and Allocation to provide guidance on organ donations [4]. Three years later, a fast channel for organ donation registration was also provided on a major e-commerce platform, in which citizens could become donor volunteers with just a few clicks [5]. Despite these efforts, the number of available organs is still below the requisite. Medical officials and professionals indicated that the plausible reasons for this shortage include the public’s insufficient awareness of and motivation for organ donation [3].

### Role of Social Media in Organ Donation

Social media plays an indispensable role in the public health landscape [6] and has the potential to leverage public engagement for organ donation. As many people are becoming accustomed to assessing health-related information online [6], social media has been widely used for communicating health information [7]. Individuals log on to social media sites to interact with each other for a variety of health issues, ranging from cancer [8] and e-cigarettes [9] to the role of virtual reality in health care [10]. Regarding organ donation, social media has also been broadly adopted for communication at the hospital, community, and grassroot levels [11].

Despite functioning as a venue for information exchange among peers, social media is a platform for promoting health regimens [6]. Information exchange usually occurs via a top-down approach and through campaigns. For example, the state health departments in the United States actively disseminate health information to citizens via Facebook and Twitter [12]. In addition, the media plays an indelible role in leveraging health topics in the social media arena. The media has been providing information on health topics such as lung cancer treatment [8] and antibiotic usage [13] on social media. The media has also been used for educational and promotional purposes for various health-related issues [2], including tobacco use [14], alcohol consumption [15], and HIV prevention [16]. With many news institutions using social media as a platform for disseminating their reports [17] and numerous news consumers habitually assessing media contents via social media [18], media campaigns on social media may play an increasingly important part in promoting public health regimens.

### Effects of Organ Donation in the Media

In the context of organ donation, the public tends to resort to the media and social media sites for information, indicating the plausibility of promoting this health behavior via media campaigns on social media. A thematic analysis of family dyads’ discussion on organ donation exemplified this predilection by revealing that the media was the most important source of information for decision making in family dyads [19]. Previous research regarding media effects also indicates that exposure to media contents may shape people’s perceptions of organ donation. For example, agenda-setting studies have shown that the media could leverage the public’s attention to the issues it covers [20]; research on framing effects has shown evidence of media’s impact on people’s attitudes [21]. Additionally, empirical studies revealed that the public would be particularly susceptible to media influence when their relevant knowledge level was low [22], which suggests that the media may exert a particularly pronounced influence on the citizens’ attitudes toward organ donation, as their understanding of organ donation is currently in a nascent state [3]. Several existing media campaigns’ effects have indeed been promising. For instance, the news reports about World Transplant Games caused noticeable surges in organ donation in many regions [23], media coverage of cornea donation boosted donation intentions in Korea [24], and media campaigns elevated eye donation awareness in India and Ethiopia [25,26].

Besides the media’s active role in promoting organ donation, the social media is an important arena for this purpose; for instance, health educators have been communicating about organ transplantation on social media sites [27]. Previous research suggested that several characteristics of social media makes it advantageous for media campaigns. Not constrained by traditional media readership of certain demographic groups, social media allows for dissemination of information to numerous citizens with various demographic characteristics and geographical locations [11,28]. Furthermore, when exposed to social media contents, people are more inclined to conduct additional information search [29,30] and express sentiments [9], which may further amplify the media campaigns’ impacts. In sum, the media effects and wide usage of social media suggest the potential of media campaigns on social media sites.

Despite the plausibility, the media’s actual effects of organ donation campaigns on social media sites remain unknown. Additionally, effective media campaigns require careful selection of media contents; however, existing knowledge about what kind of media posts are most suitable for organ donation is inadequate. Organ donation cannot be increased by merely distributing media posts, as the effect of persuasive messages may not always meet the message designers’ expectations, and some messages might even induce backfire effects [31]. For example, research regarding health messages have cautioned that the presence of smoking cues in antismoking messages would weaken their persuasive effects [32] and that the success of cancer campaigns is largely contingent on the nature of the campaigns [30]. Effectiveness of media campaigns varies by the type of issues included and the specific campaign strategies employed, and existing media campaigns for organ donation have, in general, yielded mixed results, despite a few successful cases [2].

Therefore, careful selection of media contents is crucial for organ donation campaigns, and identifying the most appropriate media contents is a critical step for this purpose. Previous studies have yielded informative findings; for example, gain rather than loss frames [33] and presentation of identifying information of organ recipients instead of donors [34] would induce more favorable reactions to organ donation. However, media posts in a natural environment are complex and nuanced, which are beyond the scope of the frameworks of these existing studies. To address this concern, media posts on organ donation on social media could provide valuable resources. An analysis of the media contents may help in profiling the existing media themes, and the themes’ effects could be assessed by approaches such as examining the repost frequencies [35]. Additionally, as the media posts and users’ reactions occur naturally, they could serve as a simulation of actual media campaigns. Spotting media themes that are most motivating for organ donation among the existing posts is also beneficial, as it allows for effective media campaigns while maintaining the existing journalistic practices.

### Research Questions

This study addressed two research questions:

1. What are the themes of the media contents regarding organ donation on social media?
2. What effects do different media themes have on people’s attitudes toward organ donation, and which themes are the most effective in promoting organ donation?

## Methods

### Data Collection

A total of 16 million Weibo users’ posts were randomly extracted from January 1 to December 31, 2017, through Weibo’s application programming interface. Weibo is the Chinese equivalent of Twitter, with an enormous amount of media posts and 37.6 million monthly active users in 2017 [36]. As such, it is a good source for observing the diffusion and effects of media posts on social media. From this dataset, 7046 posts were extracted via key word searches including combinations of “organ,” “donation,” and “shortage,” (eg, organ donation and organ shortage) and the names of particular types of organs (eg, organ, liver, and liver donation). The posts were further manually filtered to include only reposts that contain both the content of the posts and a distinguishable media source. This procedure yielded 1507 reposts, which served as the dataset for further investigation.

### Data Analysis

Primarily, we identified major themes in the media posts. The 1507 reposts were attributed to 141 distinct media posts. In line with previous research, a direct content-analysis approach was adopted to analyze these posts [37]. One researcher screened the posts and proposed a codebook for categorizing them based on media themes. Another researcher subsequently evaluated the categorizations and provided suggestions. Consensus between the two researchers was reached for all themes. Following the procedure used by a previous study [37], the two researchers then coded the first 30 pieces of the media posts, which constitutes approximately 20% of the total. Disparities in coding were discussed and resolved. If one post pertained to two themes, it was labeled according to the more salient one. Lastly, one of the researchers coded the remining posts. Media themes of the posts were identified, and all the media posts were classified according to their themes.

When browsing media posts, social media users can repost with a click and sometimes add comments along with the reposts. Adopting the methodology of a previous study [35], we used the reposting frequency of a post and the number of comments it received as proxy measures of issue awareness promoted by the post. The numbers of posts and comments received for each media theme were counted for this purpose. To gauge a particular theme’s contribution to organ donation awareness, the repost/post ratio and comment/post ratio were calculated by dividing the total number of reposts and comments pertaining to each theme by the total number of posts. For example, if 58 posts regarding “organ donation behavior” gained 295 reposts and 37 comments, the repost/post ratio would be 295/58, indicating that every post evoked approximately 5 reposts on an average, and the comment/post ratio would be 37/58, suggesting that every post induced approximately 0.64 comments.

In addition, the extent to which a media theme motivated organ donation was assessed based on the number of its comments that expressed prodonation attitudes and stated donation intentions. Using a similar procedure for the media content analysis, the comments that indicated support and intention for organ donation were coded and counted for each theme. The number of comments pertaining to a theme that showed prodonation attitudes and intentions was divided by the total number of comments pertaining to the theme, yielding a prodonation/comment ratio and intention/comment ratio, respectively. For instance, the media posts under the theme of “organ donation behavior” received 37 comments, of which 7 contained prodonation attitudes and 3 showed donation intentions, which makes the prodonation/comment ratio 7/37 and the donation/comment ratio 3/37. The results indicate that 18.92% comments pertaining to this theme expressed support for organ donation and 8.11% showed donation intentions. Finally, the media themes’ effectiveness in promoting organ donation was assessed and compared based on these indicators.

## Results

### Media Contents

Five major themes were derived from the 141 media posts: organ donation behaviors, issues and policies regarding organ donation, meaningfulness of organ donation, statistical descriptions of organ donation, and organ donation practice (Table 1).

**Table 1:** Coded themes or subthemes of media posts for organ donation.

Theme and subtheme		Number of posts	Definition	Example
Organ donation behaviors		58	The text pertains to individual donors’ or their families’ behavior related to donation or the donation process	A father donated his beloved son’s organ after the son’s death in a car accident
**Meaningfulness of organ donation**		28	The text pertains to the meaningfulness generated by organ donation	A mother heard her deceased son’s heartbeat when she met her son’s organ recipient
	Meaningfulness for donors	10	The text pertains to the meaningfulness of organ donations for the donors	A mother heard her deceased son’s heartbeat when she met her son’s organ recipient
	Meaningfulness for recipients	4	The text pertains to the meaningfulness of organ donations for the recipients	An organ recipient was grateful to the donor’s families and called them “mom and dad”
	Meaningfulness for society	14	The text pertains to the meaningfulness of organ donations for society	Organ donation extends our lives
Issues and policies regarding organ donation		28	The text pertains to the state and the problems facing organ donation or policies that address these problems	The “organ donation coordinator” was employed to help alleviate the organ donation shortage
Statistical descriptions of organ donation		21	The text pertains to the statistics of registered organ donors	In 2016, the number of registered organ donation volunteers reached 104,528 in China
Organ donation practice		6	The text pertains to the implementation and procedure of organ donation	A well-known medical professional performed an organ donation transplantation for a patient while on a visit to Beijing for a major conference

Among them, the largest proportion of the posts addressed “organ donation behaviors” (58/141, 41.13%). This theme refers to an individual donor or his family’s engagement with organ donation (eg, “The father donated his beloved son’s organ after the son passed away in a car accident”). “Issues and policies regarding organ donation” and “meaningfulness of organ donation,” each constituted 19.86% (28) of the posts. Upon close analysis, we found that three subthemes of “meaningfulness of organ donation” were observed, based on the subjects involved in organ donation: the meaningfulness of donation for the donors, recipients, and society. The remaining two themes pertained to “statistical descriptions of organ donation” (21, 14.89%) and “organ donation practice” (6, 4.26%).

Sources of the media posts were also identified and classified according to three commonly recognized media source categories in China: official media, which was responsible to the government institutions; market-oriented media, which is largely influenced by market competition; and self-media, which is initiated by individual persons or organizations [38,39]. The analysis revealed that the three types of media, in general, have taken equally active roles in disseminating organ donation information. Specifically, market-oriented media produced 34.75% of the posts, official media had a marginally lower proportion of 34.04%, and self-media produced 31.20% of the total.

### Media Effects

Of the five themes, “statistical descriptions of organ donation” had the highest repost/post ratio of 37.24%, indicating that the media posts in this theme triggered 37.24 reposts on an average, followed by “meaningfulness of organ donation,” with a repost/post ratio of 14.04%. It is also noteworthy that among its three subthemes, “meaningfulness for recipients” induced a high repost/post ratio of 27.75%. By contrast, the ratios for “organ donation practice” and “issues and policies regarding organ donation” ranked low at 1.33% and 1.04%, respectively. Regarding the comment/post ratio, “statistical descriptions of organ donation” had a value of 7.71%, which was the highest, and “organ donation behaviors” had the lowest ratio of 0.64% (Table 2).

**Table 2:** Posts, reposts, and comments across media themes.

Media themes and subthemes		Number of posts	Number of reposts	Number of comments	Repost/post ratio (%)	Comment/post ratio (%)
Organ donation behaviors		58	295	37	5.09	0.64
**Meaningfulness of organ donation**		28	393	42	14.04	1.5
	Meaningfulness for donors	10	198	24	19.80	2.4
	Meaningfulness for recipients	4	111	11	27.75	2.75
	Meaningfulness for society	14	84	7	6.00	0.5
Issues and policies regarding organ donation		28	29	4	1.04	0.14
Statistical descriptions of organ donation		21	782	162	37.24	7.71
Organ donation practice		6	8	0	1.33	0

**Table 3:** Prodonation attitudes and donation intentions expressed across media themes.

Media themes and subthemes		Number of prodonations	Prodonation/comment ratio (%)	Number of donation intentions	Donation/comment ratio (%)
Organ donation behaviors		7	18.92	3	8.11
**Meaningfulness of organ donation**		16	38.10	9	21.43
	Meaningfulness for donors	7	29.17	4	16.67
	Meaningfulness for recipients	4	36.36	1	9.10
	Meaningfulness for society	5	71.43	4	57.14
Issues and policies regarding organ donation		1	25.00	0	0.00
Statistical descriptions of organ donation		85	52.47	50	30.86
Organ donation practice		N/A^a^	N/A	N/A	N/A

^a^N/A: not applicable.

Among the 1507 reposts, 245 contained comments, of which 109 expressed prodonation attitudes and 62 comments contained clear donation intentions. These social media users showed positive attitudes toward organ donations with both straightforward claims such as “I support organ donation” and more nuanced tones such as “It shows the advancement of our society.” There were typical comments regarding organ donation intentions, such as “If I meet the requirements, I would like to donate my organs.” The media themes were compared based on the two indicators for prodonation attitudes and donation intentions. The analysis showed that “statistical descriptions of organ donation” generated the highest prodonation/comment ratio of 52.47% and intention/comment ratio of 30.86%, suggesting that 52.47% of the comments under this theme contained prodonation sentiments and 30.86% included donation intentions. “Meaningfulness of donation” had the second highest prodonation/comment ratio of 38.10% and a donation/comment ratio of 21.43%. In addition, one subtheme of “meaningfulness of donation”—“meaningfulness for society”—had a prodonation/comment ratio of 71.43% and a donation/comment ratio of 57.14%. In comparison, organ donation behaviors had both the lowest prodonation/comment ratio of 18.92% and the lowest donation/comment ratio of 8.11% (Table 3).

The effectiveness of campaigns would be further enhanced if the target audience could be profiled. Specifically, it would be informative to acknowledge people with characteristics that are more likely to transmit the organ donation posts and who are more inclined to develop prodonation attitudes after media exposure. Therefore, we took a further step to characterize the people who reposted, commenters, donation supporters, and self-reported donor volunteers by retrieving and analyzing information on their sex and age. The social media users’ characteristics in our sample were compared with those of the original 16 million social media users to ensure that the observed difference in the dataset of organ donation (ie, more young people supported organ donation than the old) was not derived from overrepresentation or underrepresentation of certain groups. The analysis showed that female individuals were more likely to comment about organ donation than male individuals and that the sex distribution of commenters in the datasets of organ donation and the dataset of 16 million users was significantly different (χ^2^_1_=23.0; *P*<.001). Moreover, individuals younger than 30 years of age were found to be more likely to repost after controlling for the age distribution in the original dataset (χ^2^_1_=54.0; *P*<.001). For donation intentions, 62 people’s sex was identifiable, and among them, more female (n=45) than male individuals (n=17) expressed willingness to become donors. Subsequently, we examined the characteristics of self-proclaimed donors via similar approaches. The analysis suggested that young people (aged less than 30 years) were more likely to donate. However, these differences were not statistically significant after controlling for the sex and age distributions in the original dataset.

## Discussion

### Principal Results

With social media and media, in general, taking increasingly active roles in the public health arena [2,6], this study empirically analyzed the effects of media for organ donation on a social media platform. The analysis identified media themes that were most effective in promoting organ donation and revealed a discrepancy between the themes that received the most media spotlight (ie, “organ donation behaviors”) and themes that were most impactful for public awareness and attitudes of organ donation (ie, “statistical descriptions of organ donation” and “meaningfulness of organ donation”). As organ donation is emerging as a concern in many countries such as China [3], the United States [34], and India [25], these findings provide practical guidance for promoting organ donation via campaigns on social media. Furthermore, this study highlighted the importance of strategic message design in promoting health regimens via campaigns on social media and explored methodological approaches to quantify the media effects by using the existing online behavioral data.

With respect to evoking the public’s donation intentions, “statistical descriptions of organ donation” was found to have a dominant advantage: 3 of 10 commenters were motivated to become donors. “Meaningfulness of organ donation” and one of its subthemes, “meaningfulness for society,” also generated a significant amount of donation intentions. These two major themes were observed in the other aspects as well (eg, prodonation attitudes). Their particular effectiveness in provoking donation intentions may stem from social norms, which could be promoted by the media, and influence people’s behavioral intentions [40,41]. The media posts of “statistical description of organ donation” might have activated a descriptive social norm, which pertains to commonly adopted behaviors [41]. In addition, “meaningfulness for society” might be connected to injunctive norms, which concerns behaviors that people think they ought to exhibit [41,42]. The notable effectiveness of “meaningfulness for society” could also be explained: As potential organ donation supporters tend to be prosocial [43], addressing social meaningfulness would further activate their motivations to donate.

In sharp contrast with these two themes’ impacts, “organ donation behaviors” had the weakest effect on promoting organ donation intentions. This result is consistent with previous experimental findings: As compared to identifying information regarding the organ recipient, presenting information of the deceased donors helped less in motivating people to register for donations, as it may induce thoughts of death rather than saving lives [34]. This study’s findings lent more empirical support to this tendency and cautioned against media’s overemphasis on the group of organ donors, which is a disadvantage not only when compared to media themes regarding organ recipients, but also when evaluated across the other themes.

Combining the findings regarding the salience and effects of the media themes, a gap was observed between the media themes that received the most media attention and those that were the most advantageous in promoting organ donation. This discrepancy indicates an unrealized potential for the media to increase organ donations; media campaigns might fulfill this goal by emphasizing donation trends and meaningfulness while curtailing the number of reports on ordinary individuals’ donation behaviors. Furthermore, the themes that are advantageous in promoting organ donation awareness, attitudes, and intentions also provide templates for message designs for future media campaigns.

An exploratory analysis was also conducted to characterize social media users who were actively involved in the issue of organ donation after exposure to the relevant media contents. The analysis of characteristics of commenters revealed patterns including one showing that female individuals more actively commented on organ donation than male individuals. This finding is consistent with previous insights that female individuals had higher level of engagement with social media [10]. No significant difference based on sex and age was observed for the group of potential donors after considering sex and age distributions in the original dataset. This might be due to the limited number of commenters who expressed willingness to donate. Future studies may further explore the characteristics of such critical groups with larger datasets.

Overall, this study examined the media effects of a health issue on social media. Previous researchers pointed out that social media has become an increasingly influential platform for health-related campaigns and a rich mine for research on health-related behaviors [6]. Echoing with these proposals, studies have analyzed social media posts’ role in health-related issues such as cancer awareness [7,30] and e-cigarette flavors [35]. Building on previous insights, this study further explored social media in the context of organ donation. Although many existing studies addressed peer interactions and public discourses regarding health issues on social media [10,35] or investigated media discourses without referring to specific platforms [44,45], this study examined the media representation of organ donation on a social media site. The analysis revealed major media themes of an important health issue and yielded practical implications for future media campaigns. Moreover, this study assessed social media posts’ influence in additional aspects. Previous research has modeled the information propagation of social media posts based on the frequency of reposts [35], whereas this study went further by incorporating the posts’ influence on attitudes and behavioral intentions. Admittedly, the operationalizations employed are not necessarily perfect; nonetheless, this approach was developed based on previous research frameworks [35]. In addition, it serves as an initial step that may inspire further methodological enhancement and suggests a route for creating more comprehensive and informative indicators for the effects on social media. Lastly, this study undertook a step to characterize the target audience for future campaigns, in response to previous researchers’ suggestions for determining the critical group [7,46]. Subsequent studies may profile the target audience using a larger sample and additional perspectives. Besides the demographic characteristics, the social media users’ preferred topics and previously expressed sentiments may also serve as aspects of their profile, and the abundant data on social media allow for investigation on this domain.

### Limitations

The quantity of the data for this analysis was not large, partly due to general insufficient awareness about organ donation [3]. Future research may track the media contents and their effects as the organ donation issue gains more importance in the media and public agenda. In addition, this study was conducted in the context of China; future studies are needed to examine the themes’ effects in other geographical and cultural contexts. This study also found it difficult to establish a causal relationship in a natural environment, and it is possible that a social media user’s expressed endorsement of organ donation originated from sources other than media exposure. Nevertheless, this bias might not be pronounced, as it is likely to exist for each of the media themes, and the conclusions were based on the themes’ relative effects rather than absolute effects. Another limitation is that not all people who reposted and developed donation intentions after the media exposure would write comments to express their thoughts; thus, the donation/comment ratio might be an underestimated indicator. On the other hand, expressing intent to donate might not be equal to the act of registering for donation, which may produce overestimation in the observation. Nevertheless, previous empirical studies have shown that behavioral intention is associated with actual behaviors [47]. In addition, assessing people’s organ donation attitudes via comments have certain advantages; compared to other research approaches like surveys, comments are helpful in avoiding socially desirable answers. For example, in a survey conducted in 2015, 89.9% respondents expressed pro-organ donation attitudes and 42.2% expressed donation intention [48]. Future studies should develop methods that allow for gauging donation intentions more accurately or comparing and quantifying the biases pertinent to different research approaches.
